# Supplementary material for: Longitudinal monitoring of prevalence and intensity of soil-transmitted helminth infections as part of community-wide mass drug administration within the Geshiyaro project in the Bolosso Sore district, Wolaita, Ethiopia
Source: PLoS Negl Trop Dis. 2022 Sep 19;16(9):e0010408. doi: 10.1371/journal.pntd.0010408 (PMC9521932; doi:10.1371/journal.pntd.0010408)
Supplement: Attachment — Submitted filename: ManuscriptReviewersComments.docx [file pntd.0010408.s007.docx]

**Summary and General Comments**

**Reviewer #1: General comments**
The study is interesting, and among other things is showing the difficulties to reach high level of compliance even in a small area under research setting and in my opinion confirm the fact that the transmission of these infections cannot be eliminated without an improvement of sanitation to a level that impede environmental contamination with human faeces.
In my opinion the paper merit publication but need a mayor review and additional analysis:

1-Despite having mentioned in page 5 the WHO cut offs, the changes in the prevalence of the different classes of intensity over time were not clearly presented in a table or in a figure. This should be done (by parasite) because will show the changes in morbidity suffered by the targeted population.

**Please see newly included Supplementary table 4 which shows the number of individuals which are designated within WHO infection intensity cut offs. This is done by species of parasite, with the results addressed Lines 12-17 Page 8**

2- since the authors have detailed information on who has been treated and who has not, it would be interesting to see the changes in prevalence and intensity of infection of the cohort of individuals that received treatment in all the MDA.

**Please see newly added supplementary table 6 which supplements figure 5. Showing numbers of individuals that have been designated; “cured”. “never infected, cured”, “Became infected” and “Remained infected” within all three treatment compliance scenarios. This is best placed as supplementary to figure 5 which is a better presentation of the information in a visual form.**

3- in the discussion (page 9) is mentioned that WASH and BBC interventions are part of the control strategy; this additional intervention should be explained in details, especially explaining what has been done in the 4 kebeles analyzed by the authors.

**Additional information has been added; see line 4-16 Page 5**

**As is outlined both in the manuscript and within the referenced geshiyaro protocol paper, the goal of the Geshiyaro project is to measure the impact and effectiveness of various WaSH, BCC and MDA methodologies on reducing infection prevalence to a level that potentially achieves transmission interruption. The WaSH and BCC methods are delivered via an additional partner in the project (World Vision Ethiopia) and is implemented in a gradual taper in terms of interventions. This means that the true impact/comparison of MDA, WaSH and BCC methods can only be truly performed at the end of the project once all strategies have been implemented across each study arm. This paper is assessing the impact of community wide MDA within these communities due to the There is currently a preprint available from the project showing the current stage of WaSH intervention effectiveness (https://www.researchsquare.com/article/rs-1266627/v1) , this is currently under review.**

Detailed comments
Main
4- Page 2 start of second paragraph.
The statement: “STH treatment guidelines target morbidity control through repeated school based deworming programmes” should be corrected. WHO currently recommends to target the 3 groups at risk: preschool children, school age children and women of reproductive age. The WHO guidelines should be cited (WHO 2017 https://www.who.int/publications/i/item/9789241550116 )

**See Line 18-19 Page 2**

5- Page 2 end of second paragraph.
“Published research indicates……. untreated individuals” the authors should mention the second factor that impede elimination of transmission that is the contamination of the environment with helminth eggs that could remain in the soil for over 10 years.

**See Line 29 – 34 page 2**

6- Page 8, first paragraph
“None of the kebeles reached the target level of 90% treatment coverage…” this is in my opinion an indication that the 90% coverage needed according modeling studies to interrupt transmission is probably not achievable in large scale programmes (and even is small research setting). this fact should be reported in the discussion

**Additional information has been added to the Discussion and conclusion of the manuscript the highlight the need to develop new tools to understand community-wide infection and a framework to improve the level of MDA coverage within a community.**

**Line 8 – 22 Page 12**

**As has been described within the manuscript, this is very much a “mid-point” in the project to assess the progress of interventions in reducing prevalence and intensity of infection. While coverage levels of 90% has not been achieved yet, MDA coverage is increasing year on year and once the final MDA rounds are delivered within the project the final coverage levels can be shown and then feasibility of transmission interruption can be reassessed.**

7- Page 9, second paragraph
“Whilst the long running ……. Interventions”
Contains one false statement: as mentioned in comment # 4 the WHO guidelines recommend to treat preschool , schools age children and WRA

**See Line 20 Page 9**

Minor
8-Page 2 line 3 I would include Ancylostoma duodenale among the main species of STH

**See Line 3 Page 2**

9-Page 2, line before last
“This create a transmission breakpoint…” I would rephrase as “This should theoretically create a transmission breakpoint…”

**See Line 38 Page 2**

10- Page 2, second paragraph
I do not consider that is correct to mention that “.. countries are considering expanding MDA….” Actually is only a group of researchers that is considering this possibility

**See Line 12 – 13 Page 3**

11-Page 6, second paragraph why the authors refer to fig 1 ? this is a table

**See Line 3 Page 5**

12-Page 6, third paragraph
Better to refer to the parasites with the correct name i.e. “A. lumbricoides” and not “Ascaris”

**See Line 22 Page 6**

**Reviewer #2: Comments**
1. Presumably this is an area that has received many rounds of historic treatment since the start of the national program in 2015 (and possibly before). Do you have any indication as what their true baseline levels are? i.e. is repeated treatment keeping infection suppressed at the current levels?

**This paper is presented as part of a wider body of work, which is the Geshiyaro project. Currently the “baseline” data presented here is part of the initial baseline survey across the entire Wolaita region across all 45 communities. This data is currently under review as a standalone publication across all sites within the study region. The baseline prevalence and intensity data presented here is taken directly from baseline survey performed across these kebeles at the start of the project. The most robust survey in terms of data accuracy took place as part of national mapping (reference in text) which did not include these kebeles as part of the survey. With this project comparing the effectiveness of disease burden reduction using various MDA strategies and BCC/WaSH at kebele level, the geshiyaro baseline survey data presented here to measure community-wide MDA effectiveness.**

**The need for accurate historical treatment and prevalence/infection data is mentioned within the discussion**

**See Lines 37-38 page 10 and 1-4 page 11**

2. Methods – you mention 45 sentinel site communities included as part of the study, but only four villages in one woreda. Do the 45 sentinel sites refer to the broader Geshiyaro project, while the four villages are for this sub-study only?

**See line 30 – 35 Page 4 and line 1 – 19 Page 5**

3. The design and impact of WASH and BCC is not mentioned. What WASH / BCC was implemented in this woreda and is it possible to disentangle the impacts of WASH, BCC, and deworming?

**Additional information detailing the WaSH and BCC interventions used as part of the Geshiyaro project within these kebeles has been added to the methods section.**

**See Line 4-17 Page 5**

**As is outlined both in the manuscript and within the referenced geshiyaro protocol paper, the goal of the Geshiyaro project is to measure the impact and effectiveness of various WaSH, BCC and MDA methodologies on reducing infection prevalence to a level that potentially achieves transmission interruption. The WaSH and BCC methods are delivered via an additional partner in the project (World Vision Ethiopia) and is implemented in a gradual taper in terms of interventions. This means that the true impact/comparison of MDA, WaSH and BCC methods can only be truly performed at the end of the project once all strategies have been implemented across each study arm. There is currently a preprint available from the project showing the current stage of WaSH intervention effectiveness (https://www.researchsquare.com/article/rs-1266627/v1) , this is currently under review.**

4. Was there a sample size conducted for the four villages / 600 individuals? Or was it convenience sampling?

**Additional information regarding the sample size calculations and longitudinal site selection has been added:**

**See line 30 Page 4**

**Additional information on the sampling within the longitudinal sentinel sites has been included.**

**See line 7-19 Page 5**

5. “From BS to Y1 the prevalence of any STH increased by 4.3%, with the change being statistically significant (P < 0.001). There was no change in prevalence, negative or positive from BS – Y1 for all species” – are these sentences contradictory?

**This has been ammended, see Line 11 – 13 Page 7**

6. “Across those individuals that were treated in both years of MDA, mean EPG dropped well below the level observed at the baseline survey (T. trichiura; 102.33 , 9 98.22, 3.22, A. lumbricoides; 583.07, 331.81, 138.71, Hookworm; 12.22, 14.92, 1.37).”It’s good to see treatment works when it’s received. The biggest challenges here (and elsewhere) is that treatment coverage is not high enough, right? Given the experience that no communities achieved the ambitious 90% coverage target even given the extra focus of the study, and that the reductions in infection were disappointing, should we reassess the feasibility of transmission interruption?

**Additional infromation has been added to the Discussion and conclusion of the manuscript the highlight the need to develop new tools to understand community-wide infection and a framework to improve the level of MDA coverage within a community.**

**Line 8 – 22 Page 12**

**As has been described within the manuscript, this is very much a “mid-point” in the project to assess the progress of interventions in reducing prevalence and intensity of infection. While coverage levels of 90% has not been achieved yet, MDA coverage is increasing year on year and once the final MDA rounds are delivered within the project the final coverage levels can be shown and then feasibility of transmission interruption can be reassessed.**

7. I’m confused by this line: “The data presented in this paper shows that compliance to MDA may be an important factor determining predisposition to high infection levels (relative to others in the community) in populations receiving preventive chemotherapy through MDA”. This is just saying that MDA reduces infection, right? Not that there’s some other mechanism. It’s the predisposition bit that’s confusing me.

**The comment has been clarified, Line 10 -15 Page 3

8. Figure 1 refers to a timeline in the text but is a table of characteristics (I think a timeline would be useful).**

Minor / Editorial
1. Introduction – is it correct to say that ALB/MEB have generally high efficacy against all species of STH?

**See Line 12-13, Page 2**

2. Introduction – I’d recommend changing ‘eradication of transmission’ to ‘elimination of transmission’

**See Line 28 Page 2**

3. It’s a minor point but decisions on treatment frequency (annual / biannual) are rarely taken on the “intrinsic transmission potential in a defined community (the magnitude of the basic reproductive number, R0)”. It’s more often taken on baseline prevalence of infection plus a range of logistical factors.

**See Line 14 – 16 Page 2**

4. Conclusion: “The study was integrated within and ongoing a national…” should probably be “The study was integrated within an ongoing national…”

**See Line 4 Page 12**

5. The 4.3% increase in STH prevalence from BL to Y1 – that’s percentage point rather than percentage, is that correct? So the percent increase is more like 13.3%

**Agreed; see ammended see line 11-13 Page 7**

6. Figures – All figure titles should be able to be read standalone. Figures 3 and 4 can’t be currently.

**See Line 6 Page 13**

**See Line 10 Page 13**

Reviewer #3: This is an interesting article on the control of soil-transmitted helminthiasis in Ethiopia. Of particular interest to me is the fact that the survey was embedded in the program and not an isolated research activity. This provides the opportunity to collect data that may not have to be tested under separate programmatic conditions as a way of validation. The abstract is also succinct and clear. The results generated should further contribute to the development of WHO guidelines by strengthening the evidence for the control and elimination of soil-transmitted helminthiasis and will be very useful to technical advisory groups including the WHO technical advisory group on soil-transmitted helminthiasis. The results, discussions, and conclusions provide critical questions that should inform future research in this field.

--------------------

PLOS authors have the option to publish the peer review history of their article ([what does this mean?](https://journals.plos.org/plosntds/s/editorial-and-peer-review-process#loc-peer-review-history)). If published, this will include your full peer review and any attached files.


If you choose “no”, your identity will remain anonymous but your review may still be made public.


**Do you want your identity to be public for this peer review?** For information about this choice, including consent withdrawal, please see our [Privacy Policy](https://www.plos.org/privacy-policy).

Reviewer #1: No

Reviewer #2: Yes: Michael French

Reviewer #3: No


Figure Files:
While revising your submission, please upload your figure files to the Preflight Analysis and Conversion Engine (PACE) digital diagnostic tool, https://pacev2.apexcovantage.com. PACE helps ensure that figures meet PLOS requirements. To use PACE, you must first register as a user. Then, login and navigate to the UPLOAD tab, where you will find detailed instructions on how to use the tool. If you encounter any issues or have any questions when using PACE, please email us at figures@plos.org.
 
Data Requirements:
Please note that, as a condition of publication, PLOS' data policy requires that you make available all data used to draw the conclusions outlined in your manuscript. Data must be deposited in an appropriate repository, included within the body of the manuscript, or uploaded as supporting information. This includes all numerical values that were used to generate graphs, histograms etc.. For an example see here: http://www.plosbiology.org/article/info%3Adoi%2F10.1371%2Fjournal.pbio.1001908#s5.
 
Reproducibility:
To enhance the reproducibility of your results, we recommend that you deposit your laboratory protocols in protocols.io, where a protocol can be assigned its own identifier (DOI) such that it can be cited independently in the future. Additionally, PLOS ONE offers an option to publish peer-reviewed clinical study protocols. Read more information on sharing protocols at https://plos.org/protocols?utm_medium=editorial-email&utm_source=authorletters&utm_campaign=protocols

__________________________________________________
In compliance with data protection regulations, you may request that we remove your personal registration details at any time. (Use the following URL: https://www.editorialmanager.com/pntd/login.asp?a=r). Please contact the publication office if you have any questions.

    
